# Application of knowledge graphs in rare disease research

**DOI:** 10.3389/fpubh.2026.1757612

**Published:** 2026-01-28

**Authors:** Yiran Fei, Huizhe Ding, Shiyuan Tong, Yibo He, Wenyu Cai

**Affiliations:** 1 The First Affiliated Hospital of Zhejiang Chinese Medical University (Zhejiang Provincial Hospital of Chinese Medicine), Hangzhou, Zhejiang, China; 2 State Key Laboratory of Medical Neurobiology and MOE Frontiers Center for Brain Science, Institutes of Brain Science, Fudan University, Shanghai, China

**Keywords:** knowledge graphs, large language models, public health, rare diseases, retrieval-augmented generation

## Abstract

Rare disease research faces significant challenges due to data sparsity and heterogeneity, leading to diagnostic delays and limited treatments. Knowledge Graphs (KGs) offer a computational solution by integrating multimodal data into structured semantic networks. This review explores the technical paradigms and applications of KGs throughout the rare disease workflow. We first describe the data foundation, focusing on standardized ontologies (e.g., HPO) and integration strategies. Subsequently, we examine core applications in elucidating pathogenic mechanisms via link prediction, enhancing clinical diagnosis through semantic reasoning, and optimizing drug repositioning using Graph Neural Networks. Notably, the review highlights the emerging integration of KGs with Large Language Models (LLMs), particularly Retrieval-Augmented Generation (RAG), to improve interpretability and precision in medical decision-making. Finally, we discuss challenges such as privacy and dynamic updates, proposing future directions like federated learning to advance the field.

## 1 Introduction

Rare disease research and clinical practice face challenges primarily due to limited patient populations and the complexity of these conditions. Despite the cumulative number of rare disease patients globally, effective treatments remain unavailable for the majority of these conditions, and patients often experience prolonged diagnostic timelines (1, 2). This issue stems largely from the fragmentation and heterogeneity of symptom information, contributing to misdiagnoses and increased medical burdens (1, 3). Concurrently, recruitment difficulties restrict traditional clinical trials, necessitating methodologies such as adaptive trial designs (4, 5). Furthermore, funding and collaboration constraints affect progress in the field (1, 5). Consequently, establishing global collaboration and data-sharing platforms using bioinformatics to extract insights from limited data represents an approach to addressing these issues (1, 3).

To address data sparsity and heterogeneity in rare diseases, Knowledge Graphs (KGs) offer a computational solution as a graph-structured knowledge representation paradigm. By integrating diverse data sources—such as genomic profiles, clinical history, and phenotypic information—KGs facilitate a framework for diagnosis and treatment, supporting medical reasoning and personalized medicine (6). Unlike traditional models characterized by computational opacity, KGs provide semantic reasoning capabilities and interpretability. Utilizing graph embedding and link prediction algorithms, they can infer potential pathogenic genes or therapeutic targets based on network topology (7). This characteristic is applicable to rare disease research where sample sizes are limited, facilitating the translation from data analysis to clinical decision support (6).

**Figure 1:**
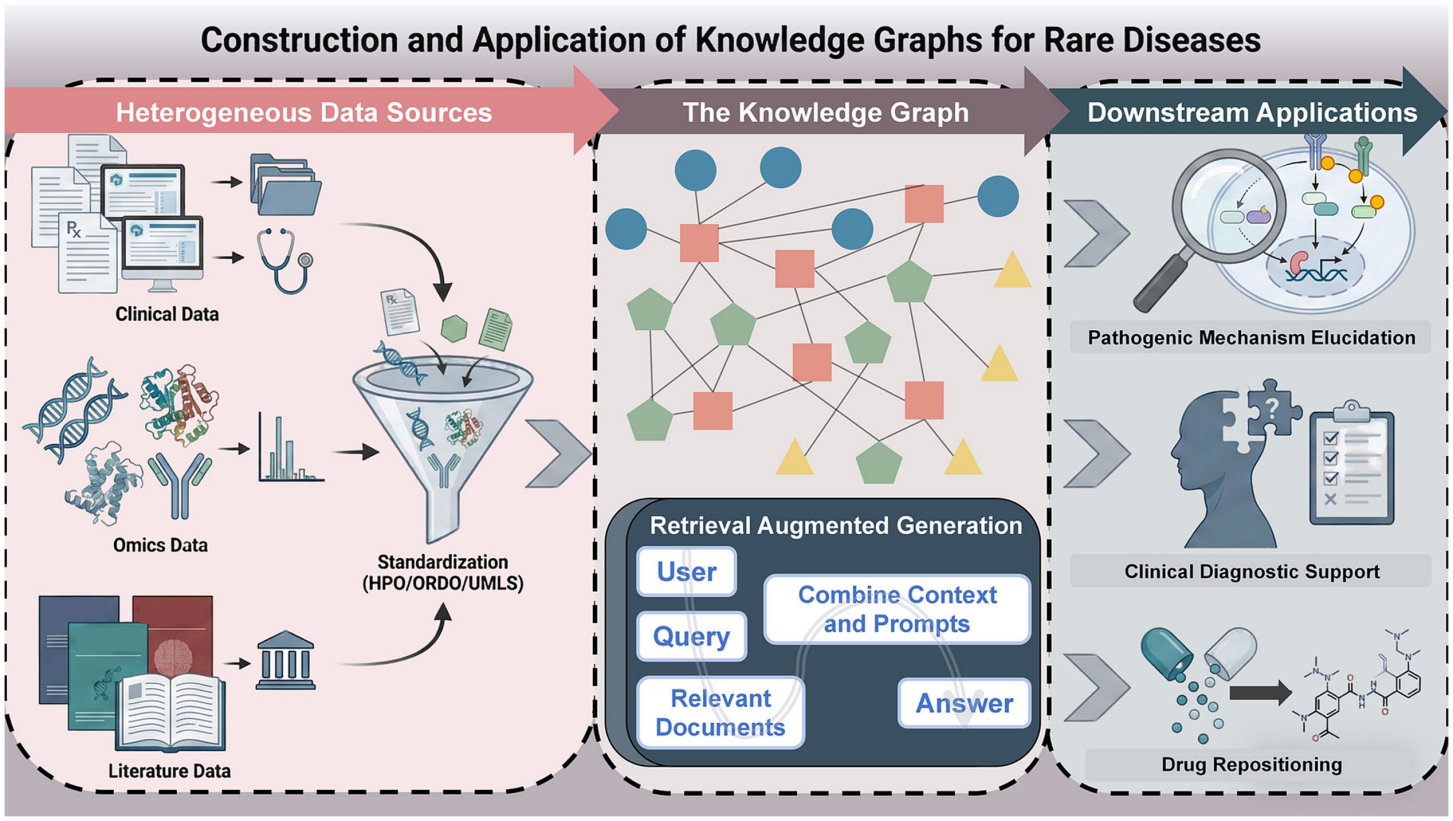
Flowchart of knowledge graph construction and application in rare disease research. (1) Construction and data basis: The process begins with the integration of heterogeneous multimodal data (clinical, omics, and literature). These data are standardized using core ontologies, such as HPO and ORDO, to construct the Rare Disease Knowledge Graph (RDKG). (2) Core applications: The structured RDKG facilitates these primary downstream tasks: elucidating pathogenic mechanisms via link prediction, providing clinical diagnostic support through semantic reasoning, and optimizing drug repositioning strategies using graph neural networks. (3) Emerging trends: The framework further integrates Retrieval-Augmented Generation (RAG), which combines Large Language Models (LLMs) with domain-specific knowledge retrieval to address hallucinations and enhance decision-making precision. HPO, Human Phenotype Ontology; ORDO, Orphanet Rare Disease Ontology; UMLS, Unified Medical Language System; RDKG, Rare Disease Knowledge Graph; RAG, Retrieval-Augmented Generation; LLMs, Large Language Models; GNNs, Graph Neural Networks.

This article aims to review the technical paradigms and applications of KGs throughout the rare disease research workflow. It first describes the data foundation for graph construction, focusing on the standardization of core ontologies and strategies for multimodal data integration. Subsequently, it examines applications in three areas: pathogenic mechanism elucidation, clinical diagnostic support, and therapeutic strategy optimization. Finally, the article explores emerging trends in integrating Large Language Models (LLMs) with KGs, specifically the potential of Retrieval-Augmented Generation (RAG) in advancing precision medicine for rare diseases (Figure 1).

## 2 Construction and data basis of rare disease knowledge graphs

### 2.1 Core ontologies and standardized vocabularies

The utility of KGs in the context of rare diseases is related to their underlying schema architecture, particularly the use of core ontologies. The Human Phenotype Ontology (HPO) supports the application of KGs in rare disease research by providing a standardized vocabulary for clinical phenotype description. Comprising over 12,000 standard terms, HPO enables the association of specific rare diseases with corresponding phenotypic abnormalities, thereby facilitating semantic alignment across different clinical data sources (8–10). This standardization supports computational algorithms based on phenotypic similarity, contributing to clinical diagnosis and genomic analysis.

KG construction also requires regulated disease classification systems and cross-domain terminology interoperability. The Orphanet Rare Disease Ontology (ORDO) serves as a reference for disease entity standardization, providing a classification system and unique identifiers for various rare diseases, which promotes interoperability with international standards such as the International Classification of Diseases (ICD) (11). To integrate different ontologies (e.g., HPO, ORDO) and biomedical concepts such as genes, proteins, and drugs, the Unified Medical Language System (UMLS) functions as a semantic pivot. Through its Concept Unique Identifiers (CUI), UMLS performs term mapping and data coordination across rare disease resources. This process helps mitigate difficulties in cross-database standardization to some extent, supporting the representation and fusion of rare disease knowledge (12). This collaborative framework of ontologies helps mitigate the sparsity and heterogeneity of rare disease data, supporting the reasoning capability and utility of KGs.

### 2.2 Multimodal data integration

The utility of Rare Disease Knowledge Graphs (RDKGs) depends on the integration of multimodal heterogeneous data, particularly from clinical, omics, and scientific literature sources. Clinical data, such as unstructured text in Electronic Health Records (EHRs), provides a source for extracting phenotypic information and understanding disease progression (13, 14). Omics data offers insights into molecular mechanisms, including gene mutations and protein interactions, which contributes to disease analysis (15, 16). Information extraction techniques, such as Named Entity Recognition (NER) and Relation Extraction (RE), are employed to identify and categorize entities and their associations, facilitating graph construction (13, 15). Subsequently, knowledge fusion strategies deduplicate and align extracted entities and relationships, improving graph consistency and completeness (17). Massive integrative frameworks like the Scalable Precision Medicine Open Knowledge Engine (SPOKE) connect millions of concepts to support precision medicine (18). Such KG integration significantly improves the discoverability of rare disease datasets by enriching source data with biological associations (19). This integration supports RDKG reasoning tasks ranging from molecular mechanism analysis to clinical decision-making.

### 2.3 Knowledge graph construction process

The construction of an RDKG involves a multi-stage process, beginning with the definition of a schema layer to establish core entities and relationships, such as those found in the HPO and Orphanet (20, 21). Subsequently, knowledge extraction techniques are employed to derive triplet structures from structured databases and unstructured literature, facilitating the organization of information (21). To address data heterogeneity, knowledge fusion and alignment processes are utilized to resolve conflicts and maintain the internal consistency of the RDKG (22). Finally, structured knowledge is stored in graph databases, where quality assessments are conducted to evaluate completeness and accuracy, supporting the reliability and reasoning capability of the RDKG in rare disease research (20, 23).

## 3 Core applications of knowledge graphs in rare disease research

### 3.1 Elucidation of pathogenic mechanisms

KGs facilitate the systematic analysis of rare disease mechanisms by integrating biomedical entities into semantic networks. The identification of pathogenic genes is frequently modeled as a link prediction task within the graph. Utilizing Knowledge Graph Embedding (KGE) techniques, such as dleMB and biokG2VEC, models represent biological features within low-dimensional vector spaces (24). By integrating HPO and Gene Ontology (GO) annotations, these methods calculate semantic distances between candidate genes and undiagnosed phenotypes, quantifying association strength and prioritizing potential pathogenic genes (25, 26). For instance, Genepredict-KG mitigates prediction bias caused by data sparsity to some extent through the fusion of multi-source data (24, 26).

Compared to the computational opacity associated with traditional deep learning models, KGs offer interpretability. Through Multi-hop Path Reasoning, KGs can trace biological interactions and infer potential causal relationships transmitting from gene mutations to clinical phenotypes. For example, frameworks such as BioKGC utilize path-based reasoning mechanisms to improve prediction accuracy while retaining logical transparency, allowing researchers to visualize paths for experimental verification (27, 28). This interpretability contributes to the reliability of results, providing mechanistic insights for clinical decision-making and drug discovery (29, 30).

At the systems biology level, KGs contribute to the analysis of molecular interaction networks and comorbidity mechanisms. By integrating Protein–Protein Interactions (PPI), metabolic, and regulatory networks, researchers can identify functional modules driving disease (31). Network topology analysis indicates that comorbidities often exhibit significant PPI overlaps and metabolic pathway associations, suggesting shared pathophysiological bases (32, 33). This systemic perspective offers insights beyond the single-gene level, contributing to the understanding of diagnostic and therapeutic strategies for complex diseases (31, 34).

### 3.2 Clinical diagnostic support and decision making

The application of RDKGs contributes to the improvement of diagnostic accuracy and efficiency, addressing challenges in rare disease diagnosis. RDKGs employ Natural Language Processing (NLP) techniques to standardize clinical phenotypes derived from Electronic Health Records (EHRs). By utilizing semantic reasoning algorithms to calculate the similarity between patient phenotypes and known rare diseases, these systems narrow the diagnostic search space to a reduced set of candidate diseases (6, 14). In this context, algorithms such as PhenoSim and Phen2Disease optimize phenotype similarity metrics to assist in prioritizing pathogenic genes based on HPO terms (35, 36). Leveraging the structured nature of KGs, these approaches aim to enhance diagnostic precision while supporting the development of personalized treatment strategies and the improvement of clinical outcomes (6, 14, 37). Research has demonstrated that even with an imperfect KG, knowledge-augmented algorithms can outperform traditional deep learning in rare disease classification (38). Clustering 3,242 rare diseases within ontology-enriched KGs using node embeddings has successfully uncovered shared pathophysiological mechanisms and drug-target connections (39).

Furthermore, the integration of Explainable Artificial Intelligence (XAI) and hybrid graph models has advanced the development of Clinical Decision Support Systems (CDSS), enhancing their applicability in clinical settings. Compared to traditional deep learning models, RDKG-based CDSSs provide transparent reasoning mechanisms, capable of delineating the logical pathways underlying diagnostic suggestions. For instance, these systems can associate patient phenotypes with genetic factors to elucidate the rationale behind a diagnosis (40, 41), thereby assisting clinicians in verifying diagnostic hypotheses and identifying potential grounds for differential diagnosis (42, 43). This transparency aids in the management of complex cases, mitigating variations in clinician expertise to a certain extent, and promoting the standardization of diagnosis and treatment (40, 43).

### 3.3 Drug repositioning and therapeutic strategies

RDKGs support drug repositioning research by integrating multidimensional drug-target-disease data. Methodologies in this field have shifted from network overlap analysis to deep learning, specifically Graph Neural Networks (GNNs). GNNs improve link prediction performance by aggregating the biological properties and topological structures of drug nodes (44, 45). Recent advancements, such as the TxGNN model, utilize Graph Foundation Models to achieve zero-shot drug repositioning, enabling the prediction of potential therapies for orphan diseases lacking prior treatment data (44, 46, 47). By utilizing large-scale biomedical KGs, these models identify therapeutic associations, addressing cost-efficiency challenges prevalent in traditional drug development (45, 46). This approach contributes to the discovery of new uses for existing drugs and has the potential to reduce development risks and costs (44, 45).

Furthermore, KGs facilitate the identification of potential therapeutic targets and the elucidation of their mechanisms of action (MoA), thereby supporting the drug discovery process. By analyzing global topological features such as node centrality, researchers can identify hub proteins linking disease phenotypes to biological pathways, suggesting potential intervention targets (48, 49). Integrating graph algorithms with XAI allows for the extraction of path-based subgraphs connecting drugs, targets, signaling pathways, and diseases, offering insights into the molecular basis of drug efficacy (50, 51). This knowledge-based reasoning has been verified in neurological disorders such as Huntington’s disease, supporting preclinical research and the investigation of therapeutic mechanisms (49). Beyond theoretical prediction, the RDKG-115 framework employed trimodal embeddings to identify candidate drugs for 115 rare diseases, providing a validated paradigm in multiple sclerosis (52). Additionally, scientific annotation KGs based on large-scale literature mining offer a systematic way to extract evidence for preclinical investigation (53). Overall, this integration of computational prediction and biological validation represents a systematic direction for drug discovery (54).

## 4 Emerging trends: applications of knowledge graphs enhanced by RAG

### 4.1 Basic concepts and medical application paradigms of RAG

RAG is an advanced framework that enhances the capabilities of LLMs by integrating external knowledge retrieval, a feature particularly beneficial for knowledge-intensive tasks. Its mechanism primarily involves a retrieval phase, which extracts relevant information from external databases, followed by a generation phase that utilizes this information to construct the output. By providing access to real-time information, this approach effectively helps mitigate key limitations associated with LLMs, such as knowledge obsolescence and hallucinations (55, 56).

In the medical domain, RAG has been applied to various scenarios, including EHR management, diagnostic support, and medical question answering. For instance, RAG can enhance EHR processing capabilities by extracting relevant patient data to generate concise summaries, thereby facilitating clinical decision support (57–59). It is also utilized to improve the accuracy of retrieving medical information from databases such as PubMed, ensuring that healthcare professionals receive timely and relevant data (60). Although the technology continues to face challenges such as retrieval noise, domain shift, and generation latency, advanced architectures like RAG+ have integrated application-aware reasoning to further enhance system interpretability and task-specific performance (61). Research indicates that RAG outperforms traditional methods in terms of precision and efficiency when generating medical responses, demonstrating significant potential to optimize healthcare delivery models (62–64).

### 4.2 Application of RAG in rare diseases

In the field of rare diseases, general-purpose LLMs often face significant deficits in domain knowledge due to data scarcity and the complexity of disease mechanisms. Integrating RAG technology with domain-specific KGs offers a strategy to address the hallucinations and knowledge gaps encountered by LLMs in this context, potentially improving diagnostic accuracy and the reliability of clinical decision-making (Table 1).

**Table 1:** Summary of knowledge graph and RAG applications in rare disease research.

Study	Methodology	Knowledge sources	Application
Song et al. (83)	Graph RAG: Hybrid structured (Cypher) and vector retrieval strategies.	FPKG (constructed from HPO).	Phenotype Diagnosis: Facial phenotype-associated genetic diseases.
Chen et al. (84)	KG-based Dynamic Few-Shot: Random walk on KG to retrieve similar cases as prompts.	Integrated KG (HPO, OMIM, Orphanet, CCRD).	Differential Diagnosis: Complex clinical scenario benchmarking.
Yang et al. (85)	Agentic RAG: Conversational agent with tool-based retrieval and multi-source fusion.	Orphanet, OMIM, GARD, Orphadata, HPO.	Consultation: Interactive diagnosis and knowledge Q&A.
Wu et al. (86)	CoT-Integrated RAG: “CoT-driven RAG” (reasoning first) strategy.	HPO database and OMIM clinical texts.	Gene Prioritization: Prediction directly from unstructured notes.
Wang et al. (87)	Corpus-based RAG: Property-specific chunking of disease reports.	NORD database.	Question Answering: Rare disease-specific QA and diagnosis.
Singhal et al. (88)	Confidence-based RAG: Selective retrieval triggered by low-confidence predictions.	Clinical guidelines and medical textbooks.	Patient Screening: Identifying undiagnosed genetic aortopathies.
Zelin et al. (66)	Knowledge-Guided RAG: Vector-based retrieval from external disease corpus.	RareDis Corpus (signs/symptoms).	Diagnostic Support: Phenotype-based disease diagnosis.
Soman et al. (89)	Context-Aware RAG: Fine-tuning models to optimize context utilization.	PubMed, NCBI GeneReviews, Patient Forums.	Patient Information: Ehlers-Danlos Syndrome queries.

CCRD, Compendium of China’s Rare Diseases, CoT, Chain-of-Thought, FPKG: Facial Phenotype Knowledge Graph, GARD, Genetic and Rare Diseases Information Center, HPO, Human Phenotype Ontology, KG, Knowledge Graph, NORD, National Organization for Rare Disorders, OMIM, Online Mendelian Inheritance in Man, RAG, Retrieval-Augmented Generation.

Existing studies suggest that combining structured domain knowledge with generative models possesses considerable application value. For example, Song et al. (65)demonstrated that integrating a Facial Phenotype Knowledge Graph (FPKG) with RAG significantly reduced temperature-induced generation variability, thereby substantially improving diagnostic consistency for rare genetic diseases. Similarly, the RaredXGPT model, which enhances ChatGPT by retrieving relevant documents from the Raredis specialized corpus, achieved diagnostic accuracy significantly superior to that of the standard ChatGPT model following the introduction of domain-specific knowledge (66). Furthermore, regarding phenotype analysis tools, the introduction of RAG technology has led to notable performance improvements. For instance, the RAG-HPO tool demonstrated superior performance over traditional HPO matching tools in key metrics such as precision, recall, and F1 score, effectively accelerating the identification of potential genetic mechanisms in rare diseases (67). Overall, RAG-enhanced Knowledge Graph applications represent a significant direction for leveraging artificial intelligence to provide high-precision and efficient diagnostic support for rare diseases, although further exploration and optimization are required to fully realize their clinical potential (68).

## 5 Discussion and outlook

### 5.1 Paradigm shift in knowledge graph-driven research

This review suggests that the application of KGs contributes to data integration capabilities and reasoning efficiency in rare disease research, facilitating a transition from local validation relying on prior knowledge to systemic reasoning based on multidimensional data. By integrating standardized ontologies such as the HPO and the ORDO, RDKGs establish a unified semantic framework, helping to manage the sparsity and heterogeneity of multimodal data.

This technological paradigm offers potential refinements to existing research and clinical processes at three levels: First, regarding pathogenic mechanism elucidation, graph embedding and link prediction methods support the prediction of potential pathogenic genes with limited prior knowledge, illustrating a progression from discrete association analysis to network-based reasoning (69, 70). Second, concerning clinical diagnostic support, computational models based on semantic similarity quantify the degree of phenotype matching, contributing to the objectivity and interpretability of diagnostic decisions (71). Finally, regarding therapeutic strategies, the application of Graph Neural Networks (GNNs) and zero-shot learning provides computational approaches aimed at addressing data scarcity in rare disease drug development (69, 71, 72). Additionally, the fusion of LLMs and KGs (including Retrieval-Augmented Generation) may improve factual consistency in generative models within medical contexts by introducing structured knowledge constraints, supporting the development of diagnostic support systems.

### 5.2 Challenges, limitations, and future

While KGs offer utility in integrating heterogeneous data, their translation into clinical practice faces technical and ethical challenges. The long-tail distribution of rare disease data and noise in EMRs limit the effectiveness of GNNs, and traditional imputation methods may be insufficient for handling such sparse data (73). Furthermore, privacy regulations, such as the General Data Protection Regulation (GDPR), restrict cross-institutional data sharing, complicating the construction of large-scale datasets (74). Addressing these challenges, future research may focus on Federated Knowledge Graphs. This paradigm supports multi-center collaborative training without exchanging raw data, facilitating adherence to privacy regulations while helping to expand sample sizes and potentially improving model generalization and accuracy (74–76). The methodological trend is shifting from simple topological analysis to multimodal representation learning and GenAI to address data sparsity (52). Future directions include user-centered trial KGs (e.g., RCTKG) to optimize clinical development by integrating trial data with standardized rare disease information (77).

Moreover, existing RDKGs are predominantly static, potentially limiting their ability to adapt to the rapid evolution of biomedical knowledge and capture recent pathogenic variants and drug mechanisms in real-time (78, 79). The lack of automated update mechanisms contributes to a lag in knowledge currency; thus, utilizing LLMs to construct Time-evolving KGs represents a notable future direction. For instance, MedkGent utilizes LLMs to construct dynamic graphs, aiming to improve the timeliness of knowledge retrieval and reasoning (79). Concurrently, to address limitations in shallow retrieval, the integration of LLMs and KGs warrants further investigation to support multi-step causal reasoning. As illustrated by Integrated Progressive Retrieval-Augmented Reasoning (IP-RAR), this fusion contributes to semantic understanding and retrieval accuracy (80). This bidirectional enhancement paradigm, combining semantic generalization with structured logical constraints, is relevant for analyzing complex pathological mechanisms and devising potential therapeutic strategies (81, 82).

## 6 Conclusion

This study reviews the applications and technical pathways of KGs in rare disease mechanism elucidation, diagnostic support, and drug development. The analysis suggests that KGs offer a framework for data integration and logical reasoning, presenting potential for identifying pathogenic associations, assisting in diagnosis, and supporting therapeutic decisions amidst challenges of data scarcity and heterogeneity. However, the clinical translation of this technology remains in an exploratory phase, constrained by issues regarding multi-source data quality, cross-institutional privacy barriers, and lags in dynamic knowledge updates. Future research warrants the exploration of technologies such as federated learning and the integration of LLMs, aiming to improve privacy security and reasoning robustness. With continued iteration and validation, KGs may provide support for evidence generation and clinical decision-making in the field of rare diseases.

## Glossary

CDG: Congenital Disorders of Glycosylation
CDSS: Clinical Decision Support System
CUI: Concept Unique Identifier
EHR: Electronic Health Record
EMR: Electronic Medical Record
FPKG: Face Phenotype Knowledge Graph
GARD: Genetic and Rare Diseases Information Center
GDPR: General Data Protection Regulation
GNN: Graph Neural Network
GO: Gene Ontology
HPO: Human Phenotype Ontology
ICD: International Classification of Diseases
IP-RAR: Integrated Progressive Retrieval-Augmented Reasoning
KG: Knowledge Graph
KGE: Knowledge Graph Embedding
LLM: Large Language Model
MoA: Mechanism of Action
NER: Named Entity Recognition
NLP: Natural Language Processing
ORDO: Orphanet Rare Disease Ontology
PPI: Protein–Protein Interaction
RAG: Retrieval-Augmented Generation
RDKG: Rare Disease Knowledge Graph
RE: Relation Extraction
UMLS: Unified Medical Language System
XAI: Explainable Artificial Intelligence

## References

[1] Wattal A Martz L. Rare diseases and their unique challenges. J Stud Res. (2024) 13:88. doi: 10.47611/jsrhs.v13i4.8088

[2] The Lancet Global Health. The landscape for rare diseases in 2024. Lancet Glob Health. (2024) 12:e341. doi: 10.1016/S2214-109X(25)00497-8 38365397

[3] Hay E Elmslie F Lanyon P Cole T. The diagnostic odyssey in rare diseases; a task and finish group report for the Department of Health and Social Care. NIHR. (2022) 2:3. doi: 10.3310/nihropenres.1115171.1

[4] Hawthorne S Valentine YA Arora A Lim F. Advancing rare disease clinical trials on an international scale: barriers and emerging solutions. MIT Sci Policy Rev. (2025) 6:4–13. doi: 10.38105/spr.27ycwhciyd

[5] Baynam G Baynam G Carrion P Houwink EJF Baynam G Carrion P . Global health for rare diseases through primary care. Lancet Glob Health. (2024) 12:e1192–9. doi: 10.1016/S2214-109X(24)00134-7, 38876765 PMC13271179

[6] Canpolat CS. Leveraging knowledge graphs for enhanced medical reasoning in personalized medicine for rare diseases. Next Fron For Life Sci AI. (2024) 8:77. doi: 10.62802/jj57vn49

[7] Sosa DN Derry A Guo M Wei EX Brinton C Altman RB. A literature-based knowledge graph embedding method for identifying drug repurposing opportunities in rare diseases. Pac Symp Biocomput. (2019) 25:463–74. doi: 10.1101/727925 PMC6937428 31797619

[8] Köhler S Øien NC Buske OJ Groza T Jacobsen JOB McNamara C . Encoding clinical data with the human phenotype ontology for computational differential diagnostics. Curr Protoc Hum Genet. (2019) 103:92. doi: 10.1002/cphg.92, 31479590 PMC6814016

[9] Groza T Köhler S Moldenhauer D Vasilevsky N Baynam G Zemojtel T . The human phenotype ontology: semantic unification of common and rare disease. Am J Hum Genet. (2015) 97:111–24. doi: 10.1016/j.ajhg.2015.05.020, 26119816 PMC4572507

[10] Bernasconi A Masseroli M. Biological and medical ontologies: Human phenotype ontology (HPO) Elsevier. *Encyclopedia of Bioinformatics and Computational Biology* (2019). doi: 10.1016/B978-0-12-809633-8.20398-1

[11] Caterina L David L Annie O Houda A Valérie S-L Marco Aurélio de C The Orphanet nomenclature of rare diseases: A standard terminology for improved patient recognition and data interoperability (2025). *arXiv* [Preprint] doi: 10.1101/2025.08.10.25333394

[12] Qian Z Dac-Trung N Eric S Anne P. Leveraging the UMLS as a data standard for rare disease data normalization and harmonization. Methods Inf Med. (2020) 59:131–9. doi: 10.1002/anie.202103557 33147635 PMC12422724

[13] Thompson W Vidmar DM Freitas JKD Pfeifer JM Fornwalt BK Chen R Large language models with retrieval-augmented generation for zero-shot disease phenotyping. arXiv. [Preprint] (2023); doi: 10.48550/arXiv.2312.06457

[14] Parikh JR Genetti CA Aykanat A Brownstein CA Schmitz-Abe K Danowski M . A data-driven architecture using natural language processing to improve phenotyping efficiency and accelerate genetic diagnoses of rare disorders. Hum Genet Genomics Adv. (2021) 2:100035. doi: 10.1016/j.xhgg.2021.100035, 34514437 PMC8432593

[15] Xing H Zhang D Cai P Zhang R Hu Q-N. Rdbridge: a knowledge graph of rare diseases based on large-scale text mining. Bioinformatics. (2023) 39. doi: 10.1093/bioinformatics/btad440 PMC10368801 37458501

[16] He YO Barisoni L Rosenberg AZ Robinson P Diehl AD Chen Y Ontology-based modeling, integration, and analysis of heterogeneous clinical, pathological, and molecular kidney data for precision medicine. bioRxiv [Preprint] (2024). doi: 10.1101/2024.04.01.587658 PMC12099421 40417545

[17] Choquet R Fonjallaz Y de Carrara A Maaroufi M Vandenbussche P-Y Dhombres F Un outil de visualisation de classifications et d'intégration de données phénotypiques et génétiques pour faciliter le codage des maladies rares [Coding rare diseases in health information systems: a tool for visualizing classifications and integrating phenotypic and genetic data] (2014). Fes, Morocco: CEUR Workshop Proceedings, 198–203.

[18] Morris JH Soman K Akbas RE Zhou X Smith B Meng EC . The scalable precision medicine open knowledge engine (SPOKE): a massive knowledge graph of biomedical information. Bioinformatics. (2023) 39:btad080. doi: 10.1093/bioinformatics/btad080 36759942 PMC9940622

[19] Braun I Hartley E Olson D Matentzoglu N Schaper K Walls R . Increased discoverability of rare disease datasets through knowledge graph integration. JAMIA Open. (2025) 8:ooaf001. doi: 10.1093/jamiaopen/ooaf001, 39926165 PMC11806703

[20] Sebastian K Leigh CC Nicole V Julius OBJ Daniel D Jean-Philippe FG . Expansion of the human phenotype ontology (HPO) knowledge base and resources. Nucleic Acids Res. (2019) 47:1018. doi: 10.1093/nar/gky1105 PMC6324074 30476213

[21] Min F Lei D Weipeng J Yuening Q, editors. DEAL: Construction of a disease-aware human cell knowledge graph from biomedicine literature (2022). Las Vegas, NV, USA: IEEE International Conference on Bioinformatics and Biomedicine (BIBM), 3804–3806. doi: 10.1109/BIBM55620.2022.9995200

[22] Dhombres F Vandenbussche P Rath A Hanauer M Olry A Urbero B . IC. Presses de l’université des Antilles et de la Guyane, (2011), 573–588.

[23] Jian Y Cong D Huilong D Qiang S Haomin L. Rdmap: a map for exploring rare diseases. Orphanet J Rare Dis. (2021) 16:101. doi: 10.1186/s13023-021-01741-4 33632281 PMC7905868

[24] Francesco G Baldomero O Janet P. Predicting gene disease associations with knowledge graph embeddings for diseases with curtailed information. NAR Genomics Bioinf. (2024) 6:lqae049. doi: 10.1093/nargab/lqae049 PMC11091931 38745993

[25] Yang L Yuchen G Xiaoyan L Chunyu W Maozu G. Pathogenic gene prediction based on network embedding. Brief Bioinform (2021);22:53. doi: 10.1093/bib/bbaa353 33367541

[26] Pittala S Koehler W. C. Deans J. R. Salinas D Bringmann M Volz K. S. Relation-weighted link prediction for disease gene identification. (2020) *ArXiv*, abs/2011.05138.

[27] Hu Y Oleshko S Firmani S Zhu Z Cheng H Ulmer M . Path-based reasoning in biomedical knowledge graphs (2024). 2024.06.17.599219. doi: 10.1101/2024.06.17.599219

[28] Annalisa M Yue H Svitlana O Samuele F Zhaocheng Z Hui C BioPathNet: Enhancing link prediction in biomedical knowledge graphs through path representation learning (2024)

[29] Liu Y Hildebrandt M Joblin M Ringsquandl M Raissouni R Tresp V. Neural multi-hop reasoning with logical rules on biomedical knowledge graphs (2021). In: Verborgh, R., et al. The Semantic Web. ESWC 2021. Lecture Notes in Computer Science, vol 12731. Cham: Springer. (2022) doi: 10.1007/978-3-030-77385-4_22

[30] Alexandre R Chloé T Michael C Ilaria T Ann N Tom L. A knowledge graph approach to predict and interpret disease-causing gene interactions. BMC Bioinformatics. (2023) 24:1–25. doi: 10.1186/s12859-023-05451-5 37644440 PMC10463539

[31] Sahrawat T. R. Systems Biology Approaches to Study Disease Comorbidities. In: Sobti, R., Sobti, A. (eds) *Biomedical Translational Research*. (2022). Springer, Singapore. doi: 10.1007/978-981-16-4345-3_6

[32] Paik H Paik H Heo H-S Ban H-J Cho SB. Unraveling human protein interaction networks underlying co-occurrences of diseases and pathological conditions. J Transl Med. (2014) 12:99. doi: 10.1186/1479-5876-12-99, 24731539 PMC4021415

[33] Lee D-S Park J Park J Kay KA Christakis NA Oltvai ZN . The implications of human metabolic network topology for disease comorbidity. Proc Natl Acad Sci USA. (2008) 105:9880–5. doi: 10.1073/pnas.0802208105, 18599447 PMC2481357

[34] Suresh NT Krishnakumar U. Topology driven analysis of protein - protein interactome for prioritizing key comorbid genes via sub graph based average path length centrality. IEEE ACM Trans Comput Biol Bioinform. (2022) 20:742–51. doi: 10.1109/TCBB.2022.3140388 34986099

[35] Xiao Y Enayati M Schaeferle GM Lanpher BC Klee EW Ngufor C. “Enhancing Patient Care in Rare Genetic Diseases: An HPO-based Phenotyping Pipeline,” 2023 IEEE International Conference on Bioinformatics and Biomedicine (BIBM), Istanbul, Turkiye (2023). 2754–2760. doi: 10.1109/BIBM58861.2023.10385978

[36] Zhai W Huang X Shen N Zhu S. Phen2Disease: a phenotype-driven semantic similarity-based integrated model for disease and gene prioritization. *Brief Bioinform*. (2023). 24:bbad172. doi: 10.1093/bib/bbad172 37248747

[37] Rima N. Natural history of rare diseases using natural language processing of narrative unstructured electronic health records: the example of Dravet syndrome. Epilepsia. (2023) 65:350–61. doi: 10.1111/epi.17855 38065926

[38] Li X Wang Y Wang D Yuan W Peng D Mei Q. Improving rare disease classification using imperfect knowledge graph. BMC Med Inform Decis Mak. (2019) 19:238. doi: 10.1186/s12911-019-0938-1, 31801534 PMC6894101

[39] Sanjak J Binder J Yadaw AS Zhu Q Mathe EA. Clustering rare diseases within an ontology-enriched knowledge graph. J Am Med Inform Assoc. (2023) 31:154–64. doi: 10.1093/jamia/ocad186, 37759342 PMC10746319

[40] Qaiser A Woo-Min J Seung Won L. Explainable AI in clinical decision support systems: a meta-analysis of methods, applications, and usability challenges. Healthcare. (2025) 13:2154. doi: 10.3390/healthcare13172154 40941506 PMC12427955

[41] Norlaili Abdul A Awais M Muhammad Deedahwar Mazhar Q Qureshi MA Wael R (2024) Explainable AI in healthcare: Systematic review of clinical decision support systems

[42] Se Young K Daeho K Minji K Hyojin K Ok-Ran J (2024) XAI-based clinical decision support system: a systematic review

[43] Egor V Alexey A. Explainable artificial intelligence in clinical decision support systems (2023).

[44] Santamaría LP Ayuso-Muñoz A Rodríguez-González A (2023) Enhancing drug repurposing through graph neural networks and link prediction. 10.1016/j.artmed.2023.102687 37925215

[45] Zhang L Li M Wang H. Graph Neural Network-Based Approaches to Drug Repurposing: A Comprehensive Survey [Preprint]. *engrxiv*. Version 2. (2025). doi: 10.31224/4410

[46] Xiong X Zhang Y Wei X Li F Guo Y Yan X Drug repurposing therapeutics prediction using hierarchical graph neural network. IEEE International Conference on Bioinformatics and Biomedicine. (2022):864–867.

[47] Papikinos T Krokidis MG Vrahatis AG Vlachakis D Vlamos P Exarchos TP. Deep learning methods for drug repurposing through heterogeneous data Elsevier BV (2024). Kunal P, Bala Chakravarthy N, Sivaraman J, editor. Academic Press, 295–313. doi: 10.1016/B978-0-443-19073-5.00005-7

[48] Zhang J Huan J (2010) Analysis of network topological features for identifying potential drug targets. Available online at: https://www.semanticscholar.org/paper/Analysis-of-Network-Topological-Features-for-Drug-Zhang-Huan/107c8b7d3c77b144f9c5c684787bc93a69e85845

[49] Menestrina L Recanatini M (2024) Knowledge graph and machine learning help the research of drugs aimed at neurological diseases. bioRxiv preprint. doi: 10.1101/2024.11.29.626076

[50] James T Hennig H. Knowledge graphs and their applications in drug discovery. Methods Mol Biol. (2024) 2716:203–21. doi: 10.1007/978-1-0716-3449-3_9 37702941

[51] Ye C Swiers R Bonner S Barrett I. A knowledge graph-enhanced tensor factorisation model for discovering drug targets. IEEE/ACM Trans Comput Biol Bioinform. (2022):1–11. doi: 10.1109/TCBB.2022.3197320 35939454

[52] Zhu C Xia X Li N Zhong F Yang Z Liu L. RDKG-115: assisting drug repurposing and discovery for rare diseases by trimodal knowledge graph embedding. Comput Biol Med. (2023) 164:107262. doi: 10.1016/j.compbiomed.2023.107262, 37481946

[53] Zhu Q Qu C Liu R Vatas G Clough A Nguyen Eth T . Rare disease-based scientific annotation knowledge graph. Front Artif Intell. (2022) 5:932665. doi: 10.3389/frai.2022.932665 36034595 PMC9403737

[54] Ozen M Emamian ES Abdi A. From data to knowledge: a mini-review on molecular network modeling and analysis for therapeutic target discovery. Arch Pharmacol Ther. (2023) 5:36–43.

[55] Zakaria H Fatima-Ezzahraa B-B Abdelhadi F (2024) SelfRewardRAG: Enhancing medical reasoning with retrieval-augmented generation and self-evaluation in large language models. 1–8. doi: 10.1109/ISCV60512.2024.10620139

[56] Genesis J. (2025) Retrieval-augmented text generation: Methods, challenges, and applications. Preprints 2025, 2025040443. doi: 10.20944/preprints202504.0443.v1

[57] Yi-feng XU. The investigation of the application of RAG technology in the field of EHR. Sci Technol Eng Chem Environ Protect. (2024) 1:9. doi: 10.61173/n7fjdj85

[58] Fnu N Deepshikha B Deepak Kumar S. Retrieval-augmented generation (RAG) in healthcare: A comprehensive review. AI. (2025) 6:226. doi: 10.3390/ai6090226

[59] He Y Chai Y Liu Y Chi J Fei Y He B . RSA-KG: a graph-based rag enhanced AI knowledge graph for recurrent spontaneous abortions diagnosis and clinical decision support. Med Res. (2025) 1:412–23. doi: 10.1002/mdr2.70039

[60] Alex-Imir T. PubMed retrieval with RAG techniques. Studies in health technology and informatics. (2024) 316:652–3. doi: 10.3233/SHTI240498 39176826

[61] Yu W Shiwan Z Zhihu W Yubo Z Xicheng Z Heyuan H RAG+: enhancing retrieval-augmented generation with application-aware reasoning. arXiv [Preprint] (2025); doi: 10.48550/arXiv.2506.11555.

[62] Rui Y Yilin N Emilia K Mingxuan L Chuan H Danielle SB Retrieval-augmented generation for generative artificial intelligence in medicine arXiv [Preprint] (2024). doi: 10.48550/arXiv.2406.12449

[63] Shailja G Rajesh R Sandeep S A comprehensive survey of retrieval-augmented generation (RAG): Evolution, current landscape and future directions arXiv [Preprint] (2024). doi: 10.48550/arXiv.2410.12837

[64] Yuhe K Liyuan J Kabilan E Hairil Rizal A Nan L Alex Tiong Heng S Development and testing of retrieval augmented generation in large language models - a case study report. arXiv [Preprint] (2024); doi: 10.48550/arXiv.2402.01733.

[65] Song J Xu Z He M Feng J Shen B. Graph retrieval augmented large language models for facial phenotype associated rare genetic disease. NPJ Digit Med. (2025);8:543. doi: 10.1038/s41746-025-01955-x. PMC12375027 40849403

[66] Zelin C Chung WK Jeanne M Zhang G Weng C. Rare disease diagnosis using knowledge guided retrieval augmentation for ChatGPT. J Biomed Inform. (2024) 157:104702. doi: 10.1016/j.jbi.2024.104702, 39084480 PMC11402564

[67] Garcia BT Westerfield L Yelemali P Gogate N Rivera-Muñoz EA Du H . Improving automated deep phenotyping through large language models using retrieval-augmented generation. Genome Med. (2025) 17:521. doi: 10.1186/s13073-025-01521-w, 40826123 PMC12359922

[68] Amugongo LM Mascheroni P Brooks S Doering S Seidel J. Retrieval augmented generation for large language models in healthcare: a systematic review. PLOS Digit Health. (2024) 4:e0000877. doi: 10.1371/journal.pdig.0000877 PMC12157099 40498738

[69] Ali S Jacques F. DNA language model and interpretable graph neural network identify genes and pathways involved in rare diseases. arXiv [Preprint] (2024).

[70] Tengyue H Xuanyu L Ao L Xin-yue Z Zhi Y. Research and practice on construction of medical data knowledge graph (2025) 2:11–9. doi: 10.70693/itphss.v2i8.1225

[71] Yunfan C Haodong H Ziqian X. Applications of artificial intelligence in rare disease research: a technical framework and future prospects. Trans Comput Sci Intellig Syst Res. (2025) 9:429–37. doi: 10.62051/yncd5x09

[72] Yinbo L Grigor'eva N Siqi W Jinming W Hesong Q Sijun M Graph network-based analysis of disease-gene-drug associations: zero-shot disease-drug prediction and analysis strategies. Biorxiv [Preprint] (2024). doi: 10.1101/2024.12.30.630746

[73] Viñas R Zheng X Hayes J. A graph-based imputation method for sparse medical records. arXiv [Preprint] (2021). doi: 10.48550/arXiv.2111.09084

[74] Fu X King I. FedHGN: a federated framework for heterogeneous graph neural networks. arXiv [Preprint] (2023). doi: 10.48550/arXiv.2305.09729

[75] Dou ZL Bai G Han Z Li Y. PFGL-net: a personalized federated graph learning framework for privacy-preserving disease prediction. J Artif Intell Res. (2025) 2:12–23. doi: 10.70891/jair.2025.080005

[76] Yang H Wang M Dai L Wu Y Du J. Federated graph neural networks for heterogeneous graphs with data privacy and structural consistency. 2025 5th International Conference on Computer Science and Blockchain (CCSB) Shenzhen, China (2025).

[77] Yang JP Leadman D Ballew RM Sid E Xu Y Mathe EA . User centered rare disease clinical trial knowledge graph (RCTKG). Stud Health Technol Inform. (2024) 310:94–8. doi: 10.3233/SHTI230934, 38269772 PMC11806939

[78] Cao L Sun J Cross A. AutoRD: an automatic and end-to-end system for rare disease knowledge graph construction based on ontology-enhanced large language models (preprint). JMIR Med Inform. (2024) 12:e60665. doi: 10.2196/60665 PMC11683654 39693482

[79] Zhang D Wang Z Li Z-Z Yu Y Jia S Dong J Medkgent: a large language model agent framework for constructing temporally evolving medical knowledge graph. arXiv. [Preprint] (2025) doi: 10.48550/arXiv.2508.12393.

[80] Feng Y Wang J He R Zhou L Li Y A retrieval-augmented knowledge mining method with deep thinking LLMs for biomedical research and clinical support. arXiv [Preprint] (2025). doi: 10.48550/arXiv.2503.23029 PMC12448786 40971592

[81] Li D Xu F. Synergizing knowledge graphs with large language models: a comprehensive review and future prospects. arXiv [Preprint] (2024) doi: 10.48550/arXiv.2407.18470

[82] Baldazzi T Bellomarini L Sallinger E. Knowledge graph-based reasoning in large language models. Front Artif Intell Appl. (2025). IOS Press. doi: 10.3233/FAIA250219

[83] Song J Xu Z He M Feng J Shen B. Publisher correction: graph retrieval augmented large language models for facial phenotype associated rare genetic disease. NPJ Digit Med. (2025) 8:604. doi: 10.1038/s41746-025-02017-y, 41062673 PMC12508205

[84] Chen X Mao X Guo Q Wang L Zhang S Chen T, editors. RareBench: can LLMs serve as rare diseases specialists? Proceedings of the 30th ACM SIGKDD conference on knowledge discovery and data mining (2024).

[85] Yang J Shu L Duan H Li H. RDguru: a conversational intelligent agent for rare diseases. IEEE J Biomed Health Inform. (2025) 29:6366–78. doi: 10.1109/JBHI.2024.3464555, 39298307

[86] Wu D Wang Z Nguyen Q Wang K. Integrating chain-of-thought and retrieval augmented generation enhances rare disease diagnosis from clinical notes. arXiv [Preprint] (2025). doi: 10.48550/arXiv.2503.12286

[87] Wang G Ran J Tang R Chang C-Y Chuang Y-N Liu Z Assessing and enhancing large language models in rare disease question-answering. arXiv [Preprint] (2024). doi: 10.48550/arXiv.2408.08422

[88] Singhal P Li Z Yang Z Nandi T Morse C Rodriguez Z . Leveraging open-source large language models to identify undiagnosed patients with rare genetic aortopathies medRxiv [Preprint] (2025) doi: 10.1101/2025.09.05.25333227

[89] Soman K Langdon A Villouta C Agrawal C Salta L Peetoom B Zebra-llama: a context-aware large language model for democratizing rare disease knowledge. arXiv [Preprint] (2024). doi: 10.48550/arXiv.2411.02657

